# Applications of fractured continuum model to enhanced geothermal system heat extraction problems

**DOI:** 10.1186/2193-1801-3-110

**Published:** 2014-02-24

**Authors:** Elena A Kalinina, Katherine A Klise, Sean A McKenna, Teklu Hadgu, Thomas S Lowry

**Affiliations:** Sandia National Laboratories, P.O. Box 5800, Albuquerque, NM 87185 USA; IBM Research, Smarter Cities Technology Centre, Bldg. 3, Damastown Industrial Estate, Mulhuddart, Dublin 15, Ireland

**Keywords:** Geothermal reservoir simulation, Enhanced geothermal systems, Heat extraction: Fracture network, Anisotropic permeability, Fractured continuum model, Geostatistical simulations

## Abstract

This paper describes the applications of the fractured continuum model to the different enhanced geothermal systems reservoir conditions. The capability of the fractured continuum model to generate fracture characteristics expected in enhanced geothermal systems reservoir environments are demonstrated for single and multiple sets of fractures. Fracture characteristics are defined by fracture strike, dip, spacing, and aperture. The paper demonstrates how the fractured continuum model can be extended to represent continuous fractured features, such as long fractures, and the conditions in which the fracture density varies within the different depth intervals. Simulations of heat transport using different fracture settings were compared with regard to their heat extraction effectiveness. The best heat extraction was obtained in the case when fractures were horizontal. A conventional heat extraction scheme with vertical wells was compared to an alternative scheme with horizontal wells. The heat extraction with the horizontal wells was significantly better than with the vertical wells when the injector was at the bottom.

## Introduction

The major objective of this work is to demonstrate the applications of the fractured continuum model (FCM) to common enhanced geothermal systems (EGS) reservoir conditions. In our previous work (Kalinina et al. 2011, Kalinina et al. 2012a, b) we conducted a number of reservoir simulations assuming homogeneous and heterogeneous reservoir conditions. This work demonstrates the importance of natural fracture properties on heat extraction.

The FCM approach was selected because it is computationally effective compared to the discrete fracture approach. This is especially important when the fracture network is very complex and the scale of interest is very large, as it is in the case of EGS. A number of different techniques were proposed to translate individual fracture properties into a continuum model (Botros et al. 2008, McKenna and Reeves, 2006, Reeves et al. 2008). The method that we used is an extension of the method described in McKenna and Reeves (2006) and (Reeves et al. 2008). The method is based on mapping the fracture properties into a continuum model regular grid. The permeability tensor calculated as a part of this mapping allows for preserving the properties of fracture network. A similar approach applied to a dual porosity continuum model is proposed in Hao et al. (2013). Hao et al. (2013) also demonstrated the ability of the FCM to accurately simulate heat transport in fractured systems by comparing the modeling results obtained with FCM and a discrete fracture model. Our goal was to modify the FCM approach to better represent the conditions typical for EGS, such as continuous fracture features and high variability of fracture density with depth.

To describe the common EGS conditions, we compiled data from the literature on natural fractures in granitic rocks (Kalinina et al. 2012a, b). These data suggested that a typical EGS reservoir may have 2 to 4 sets of sub-vertical fractures with different dips and orientations. Each set may have different fracture spacing and aperture. The fracture density may also change with depth, and repeating intervals with high and low fracture densities are very common. To incorporate these data in the reservoir simulations, we extended the previous FCM designed for vertical fractures (McKenna and Reeves 2006; Kalinina et al. 2012a, b). The new FCM is described in (Kalinina et al. 2012a, b) and incorporates fully three-dimensional representations of anisotropic permeability, multiple independent fracture sets, and arbitrary fracture dips and orientations.

The goal of this work is to demonstrate the capabilities of the FCM to generate different types of fractures, to evaluate the impacts of these different fracture types on heat extraction, and to compare alternative heat extraction schemes.

## Method

The FCM maps the permeability of discrete fractures onto a regular grid using a continuum approach. This approach defines fracture sets using the strike, dip, aperture, and spacing of parallel fractures in the system. The purpose of the FCM is to generate multiple permeability fields using field observations of fracture set strike, dip, aperture, and spacing. Both, natural fractures or fractures created with hydro-fracturing can be represented.

Parallel plate flow methods, originally presented by Snow (1968,1969), were extended to include multiple fracture sets at arbitrary fracture orientation following the method developed by Chen et al. (1999).

The continuum approach computes permeability tensors for each grid cell in the model domain. For one fracture set, the permeability tensor is defined in Eq. (1) (Chen et al. 1999).1

where *k*_*ij*_ is the permeability tensor in the *i* = *x,y,z* and *j* = *x,y,z* directions, *b* is fracture aperture, *d* is fracture spacing, and *n*_*1,2,3*_ is the unit normal to the fracture plane in the *x*, *y*, and *z* direction, respectively.

The unit normal components to the fracture plane are defined in Eq. (2).2a2b2c

where *α* = 90° - dip and *ω* = strike - 90°.

In the case of multiple fracture sets, the permeability tensor can be computed by summing the permeability tensors for individual fracture sets as follows.3

where *N* is the number of fracture sets and *k*_*ij*_^*m*^ is defined by Eq. (1). The summation assumes that the total porosity within a grid-cell changes very little, which is valid for most EGS conditions. Note that only *k*_xx_^*^, *k*_yy_^*^, and *k*_zz_^*^ components of the permeability tensor are used in the heat transport model.

The *k*_*xx*_^*m*^, *k*_*yy*_^*m*^ and *k*_*zz*_^*m*^ values are calculated for each grid block based on the fracture aperture, spacing, strike, and dip probability distributions that are defined for the fracture set *m*. Four probability distributions are currently incorporated: normal, log-normal, exponential, and power law. However, any other distribution can be easily added.

SGSIM (Deutsch and Journel, 1998) is used to generate a multiGaussian field of spatially correlated numbers. At each grid point, the simulated value can be considered a Gaussian deviate transformed to a uniform deviate. At each grid point, the uniform deviate is then used to draw from a fracture parameter distribution (i.e., aperture, spacing, strike, and/or dip). This is essentially the probability field approach to stochastic simulation. Here, there are no conditioning data, so all distributions for the chosen fracture parameter are identical across the domain and the spatial correlation in the simulated uniform deviates is translated to the fracture parameter. The orientation and magnitude of the spatial correlation can be specified to create continuity in similarly valued parameters in certain directions. Individual fracture sets can utilize both correlated/uncorrelated properties (e.g., correlated spacing but uncorrelated apertures).

As noted in literature search, fracture properties often change with depth. Different fracture parameters can be assigned to different model regions to modify fracture density with depth. By adjusting the fracture aperture and spacing with depth, zones of high and low fracture density can be created.

The resulting *k*_xx_^*^, *k*_yy_^*^, and *k*_zz_^*^ values for each grid cell calculated using Eq (1) through Eq. (3) are written in the format required for the heat transport simulations with the Finite Element Heat and Mass Transfer code FEHM (Zyvoloski et al. 1997).

### Applications

Our goal is to consider FCM applications to common EGS conditions and to examine heat extraction as a function of these conditions. These conditions include the following:

i. Continuous fractured features.
ii. Fractures with different spatial orientation
iii. Multiple fracture sets.
iv. Depth intervals with different fracture density.

The discussion of these applications is provided below. The permeability fields were generated for a modeling domain measuring 300 m by 300 m in the horizontal directions and 200 m in the vertical direction using a constant orthogonal grid with a grid cell size of 5 m by 5 m by 5 m.

### Continuous fractured features

Natural fractures in granite often form multiple sets of parallel fractures that extend to significant distances. Fractures created with hydro-fracturing also may extent to a few tenths of meter. Consequently, the FCM needs to have a capability to generate continuous fractures. Our first goal was to define the fracture parameters or the combination of fracture parameters that allows for generating continuous fracture sets.

As discussed above, the spatial correlation in fracture orientation can be introduced via spatially correlated random numbers, which can then be used to define any combination of fracture parameters. We first used the spatial correlation for one of the fracture parameters while keeping the other three parameters uncorrelated. We then considered different combinations of the spatially correlated fracture parameters.

Based on our experiments we concluded that fracture spacing is the major parameter controlling the spatial continuity of the fractures. The importance of the fracture aperture and spacing is clear from Eq. (1). However, using the spatial correlation for the aperture alone did not allow for generating continuous features.

The following approach to generating continuous fractured features is recommended. To generate fractures that extend through multiple grid cells, fracture spacing is correlated in the direction of strike and dip. The strike, dip, and aperture can either use the same correlated field as the spacing or a random number generator to assign the value for each cell. The probability distribution function for strike and dip should be aligned with the spatial correlation for spacing. For example, if the spatial correlation for spacing has a strike of 45° and a dip of 0°, the mean value for strike should be 45° and the mean value for dip should be 0°. In this way, fractures extend through multiple grid cells with similar properties. The correlation length used for spacing controls the length of that fracture set.

**Figure 1:**
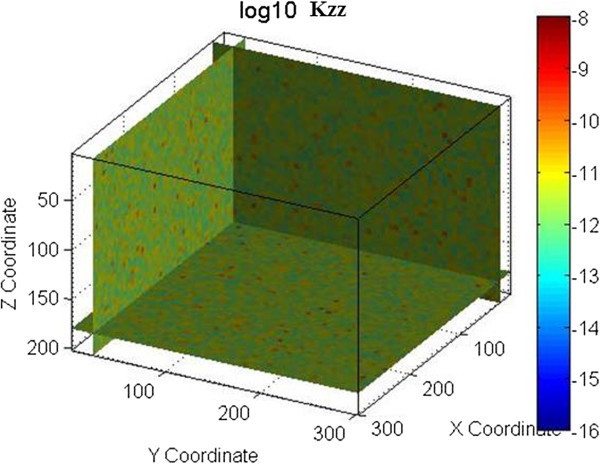
**Permeability field generated using spatial correlation in fracture aperture.** Permeability is in m^2^.

**Figure 2:**
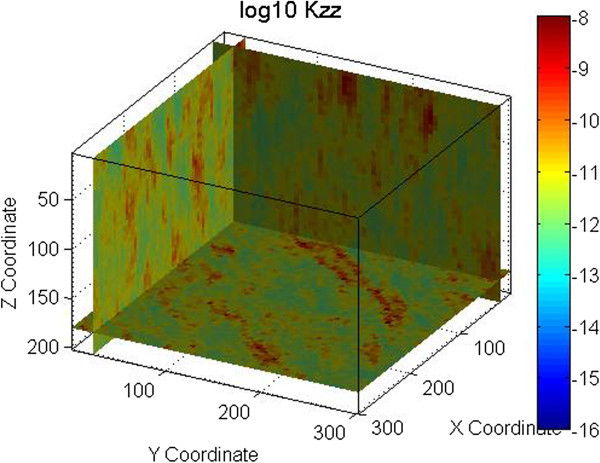
**Permeability field generated using spatial correlation in fracture spacing.** Permeability is in m^2^.

An example is shown in Figures 1 and 2 for one fracture set with a mean strike of 135° and a mean dip of 90°. A normal distribution with a mean of 3.35×10^-4^ m (0.335 mm) and a standard deviation of 3.35×10^-5^ m (0.0335 mm) was used for the fracture aperture. An exponential distribution with a mean of 1.0 m, minimum of 0.001 m and maximum of 10 m was used for the fracture spacing. The permeability field shown in Figure 1 was obtained using spatial correlation in generating the grid cell fracture apertures, while in Figure 2 spatial correlation was used in generating the grid cell fracture spacing. Figures 1 and 2 demonstrate that the spatial correlation in fracture aperture does not produce continuous features while spatial correlation in fracture spacing does. Note that in this example the search radius for SGSIM was defined to be half of the modeling domain size (150 m).

### Fractures with different spatial orientation

In our previous work (Kalinina et al. 2012a, b), we demonstrated the importance of vertical anisotropy in permeability for heat extraction using a homogeneous anisotropic reservoir as an example. We showed that heat extraction can be significantly improved in the case when horizontal reservoir permeability is a few orders of magnitude greater than the vertical permeability. We also concluded that vertical fractures (vertical anisotropy is equal to 1) are the worst case for heat extraction because they allow cold water to sink to the bottom of the reservoir.

In this work we examine fractures with different dips, evaluate the average vertical anisotropy as a function of fracture dip, and demonstrate the impacts of spatial orientation on heat extraction.

**Figure 3:**
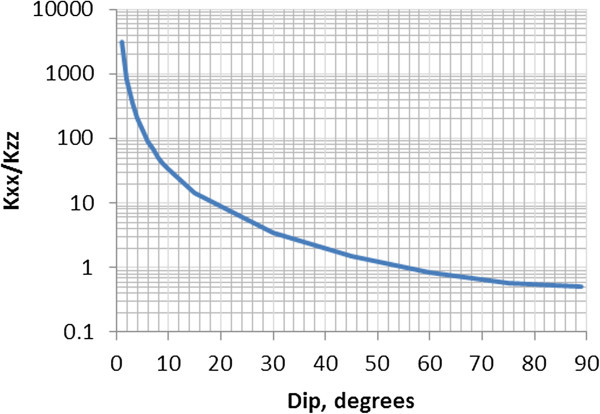
**Vertical anisotropy as a function of fracture dip.**

The average ratio of horizontal and vertical permeability (k_xx_/k_zz_ or k_yy_/k_zz_) as a function of mean fracture dip can be calculated from Eq. (1) and Eq. (2) using the mean fracture parameters. Note that in the case when the strike is either 45° or 135°, k_xx_ = k_yy_ and k_xx_/k_zz_ = k_yy_/k_zz_. Figure 3 demonstrates k_xx/_k_zz_ as a function of fracture dip for the case with the mean fracture strike of 135°, mean aperture of 3.35×10^-4^ m, and mean spacing 1.0 m. This functional relationship is very similar to the other cases with different mean fracture spacing and aperture values.

The vertical anisotropy is 2 orders of magnitude or greater (Figure 3) only in the case of sub-horizontal fractures with a dip of less than 6°. In the case of sub-vertical fractures (dip greater than 60°), the vertical permeability is greater than the horizontal permeability. Note that natural fractures in deep granite rocks are mostly sub-vertical, which leads to insignificant anisotropy in vertical direction.

### Heat extraction for different fracture settings

To investigate in impact of various fracture properties on heat extraction simulations, the following cases were considered:

i. One set of close to vertical fracture (strike of 135°, dip 85°)
ii. One set of close to horizontal fractures (strike of 135°, dip 2°)
iii. One set of fractures with dip of 10° (strike of 135°)
iv. Two sets of close to horizontal nearly orthogonal fractures (strike of 130° and 45°, dip 2°)
v. Different fracture sets within the different depth intervals

The close to horizontal fractures are not common in granite rocks at large depth. However, these fractures might be created with hydro-fracturing. This was one purpose for considering the close to horizontal fractures in the heat extraction simulations. Another purpose was to evaluate the effects of vertical anisotropy, which is at its maximum value for horizontal fractures.

**Figure 4:**
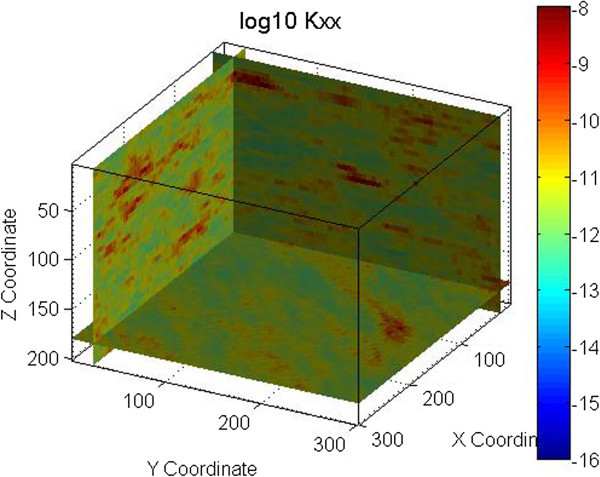
**Permeability field for one set of horizontal fractures.** Permeability is in m^2^.

**Figure 5:**
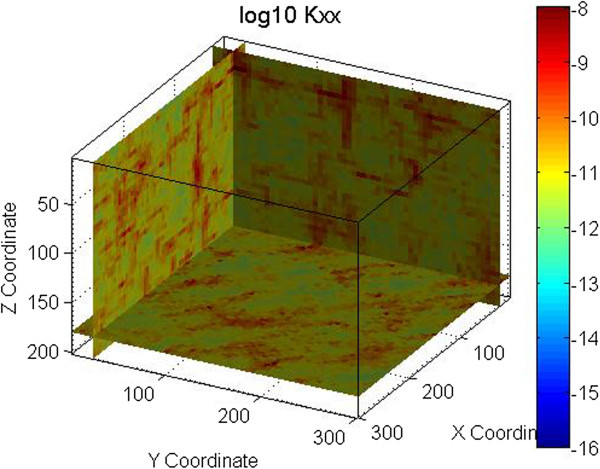
**Permeability field for two sets of horizontal fractures.** Permeability is in m^2^.

**Figure 6:**
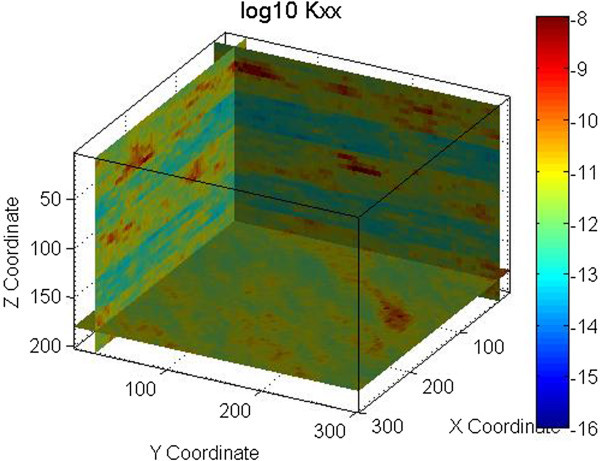
**Permeability field for one set of horizontal fractures with different aperture and spacing within different depth intervals.** Permeability is in m^2^.

The permeability fields for these examples were generated using FCM as defined by Eq. 1 through 3. Figure 2 shows the permeability field generated for the vertical fracture case. Figures 4, 5, and 6 show the permeability fields for the other cases, except the case with a dip of 10°, which is similar to the case with horizontal fractures. These figures demonstrate that FCM is capable of generating continuous fractured features for a range of conditions.

The thermo-hydrology simulations in a fractured reservoir were conducted using the numerical computer code FEHM (Zyvoloski et al. 1997). FEHM is a control volume finite element code for simulating subsurface non-isothermal multi-phase multi-fluid heat and mass transfer developed at Los Alamos National Laboratory (LANL). The code has been extensively used for modeling geothermal systems and groundwater flow and transport, including large scale projects, such as performance assessment of Yucca Mountain Project and Environmental Remediation of the Nevada Test Site. The governing equations for the conservation of mass and energy implemented in FEHM are provided below. More details can be found in (Zyvoloski et al. 1997).

Conservation of mass for water is described as:4

where the mass per unit volume, *A*_*m*_, is given by:5

and the mass flux,  is given by:

where, ϕ is the porosity of the matrix, *S* is saturation, *ρ* is density, *η* is the concentration of the noncondensible gas and is expressed as a fraction of the total mass,  is velocity, and the subscripts *v* and *l* indicate quantities for the vapor phase and the liquid phase, respectively. Source and sink terms are represented by the term *q*_*m*_*.*

Conservation of fluid-rock energy is described as:6

where the energy per unit volume, *A*_*e*_, is given by:7

and the energy flux,  is given by:8

The subscript *r* refers to the rock matrix, *u*_*r*_, *u*_*v*_*,* and *u*_*l*_ are specific internal energies, c_*pr*_ is the specific heat, h_v_, and *h*_*l*_ are specific enthalpies, *K* is an effective thermal conductivity, *T* is the temperature, and *q*_*e*_ is the energy contributed from sources and sinks.

It is assumed that Darcy’s Law applies to the movement of each phase and the velocity can be expressed as:9a9b9c

where *k* is the permeability, *R*_*v*_, and *R*_*l*_*,* are the relative permeabilities, *p*_*v*_ and *p*_*l*_ are viscosities, *P*_*v*_ and *P*_*1*_ are the phase pressures, *P*_*cap*_ is the capillary pressure and ,  represents the acceleration due to gravity. The equations are shown for an isotropic medium, though this restriction does not exist in the computer code.

We used the FEHM capability to simulate heat and mass transfer with pressure and temperature dependent properties required for modeling a geothermal system. FEHM incorporates constitutive relationships that describe pressure and temperature dependent fluid and gas properties, relative permeabilities and capillary pressures, stress dependencies, and reactive and sorbing solutes.

**Figure 7:**
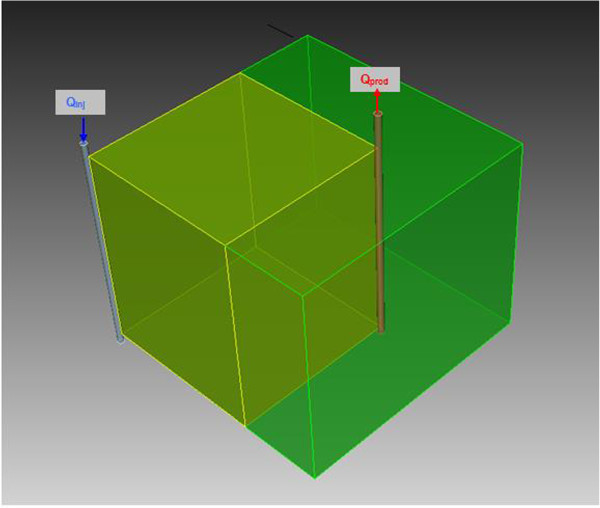
**Conceptual representation of the reservoir numerical model.**

The considered model (Figure 7) represents ¼ of the reservoir. This was done to take advantage of a symmetry introduced by a 5-point injection scheme in which there is one injection well in the middle and 4 production wells in the corners. The model is 300 m by 300 m in the horizontal directions and 200 m in the vertical direction. The grid block size is 5 m by 5 m by 5 m. The total production rate was 30 kg/s. The injection well was located in the corner of the modeling domain at *x* = 0, *y* = 0 m. The production well was located 417 m away from the injection well along the model diagonal (*x* = 300 m, *y* = 300 m). The fracture trend was aligned with the model diagonal connecting the injection and the production well. The initial reservoir temperature was 225°C and the injection temperature was 80°C.

The pre-injection boundary conditions are hydrostatic pressures corresponding to the depth to the reservoir top (4 km) and constant temperature on the vertical sides of the model, constant pressure and temperature on the top of the model, and zero flux on the bottom of the model. Zero flux conditions are specified across all the boundaries during the injection. Injection is implemented using a fixed injection mass-flow rate. The production well is implemented by specifying a fixed bottom-hole pressure in the production well.

**Figure 8:**
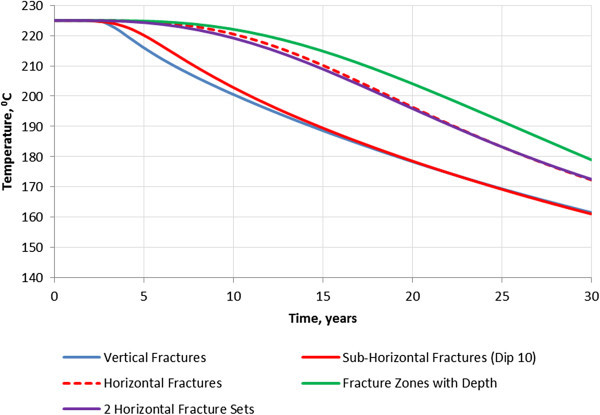
**Average temperature in the production well for different fracture settings.**

Figure 8 shows the results of the heat simulations for the different fracture settings in terms of average temperature in the production well as a function of time from the beginning of injection.

Figure 8 demonstrates that heat extraction in the case with fractures dipping 10° (k_xx_/k_zz_ = 33) is similar to the case with the vertical fractures. The temperatures at the end of the injection are the same in both cases. This result is consistent with our previous work in which we considered a homogeneous anisotropic reservoir (Kalinina et al. 2012a, b).

The heat extraction in the case of horizontal fractures (k_xx_/k_zz_ =3,119) is significantly better than in the case of vertical and sub-horizontal (dip 10°) fractures. The temperature begins to decrease 5 years later and is 10 °C higher by the end of injection. There is a small difference in performance (temperature histories) between the case with one set of horizontal fractures and two sets of horizontal fractures.

**Figure 9:**
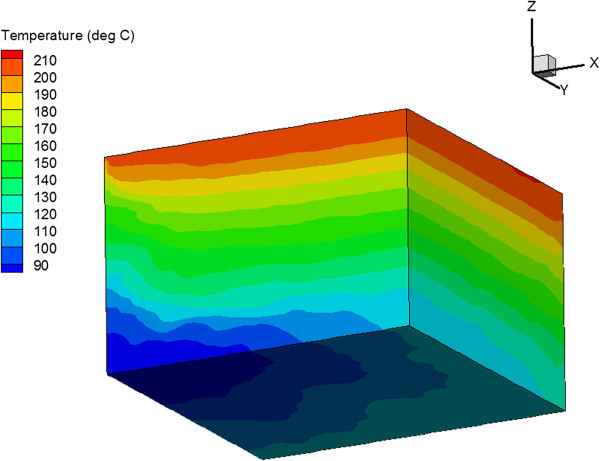
**Temperature distribution after 30 years of injection for reservoir with one set of vertical fractures.**

**Figure 10:**
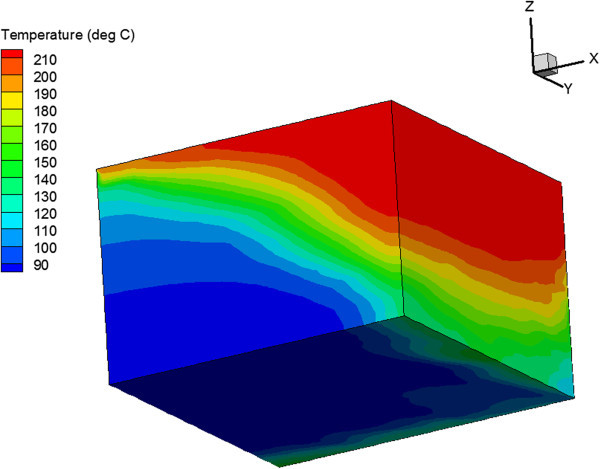
**Temperature distribution after 30 years of injection for reservoir with one set of horizontal fractures.**

The temperature distribution at the end of the simulation is shown in Figure 9 for the vertical fracture case and in Figure 10 for the horizontal fracture case. These figures show that a larger reservoir volume is in the heat exchange in the horizontal fracture case. This leads to slower cooling and higher production temperatures.

**Figure 11:**
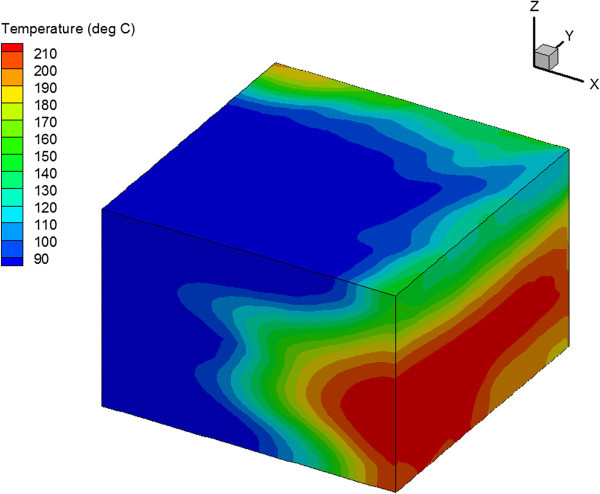
**Temperature Distribution after 30 Years of Injection for Reservoir with Different Fracture Density in Different Depth Intervals.**

The heat extraction is also improved in the case when there are intervals with high density and low density horizontal fractures compared to the case with horizontal fractures with constant density. The production temperature after 10 years is 3° to 6° higher. However, the higher temperature were only obtained in the case when the vertical permeability in the low fracture density intervals is 2 orders of magnitude lower than in the high fracture density intervals. Otherwise, the production temperatures are similar in both cases. The intervals with low fracture density and low vertical permeability create additional vertical anisotropy. As a result, the temperature front is spread over a larger reservoir volume, which improves heat exchange and lead to slower cooling and higher production temperatures. The temperature distribution at the end of injection for this case is shown in Figure 11.

### Alternative heat extraction schemes

Our simulations for the different fracture settings demonstrated that the best heat extraction conditions are in the case of horizontal fractures. Even gently dipping fractures impact the heat extraction in a negative way and make it similar to the worst case of sub-vertical fractures. Because the natural fractures in granite rock are sub-vertical, the extraction of heat with vertical wells will likely be limited.

One solution to this problem might be to use a different heat extraction scheme. An example of an alternative scheme is considered below.

**Figure 12:**
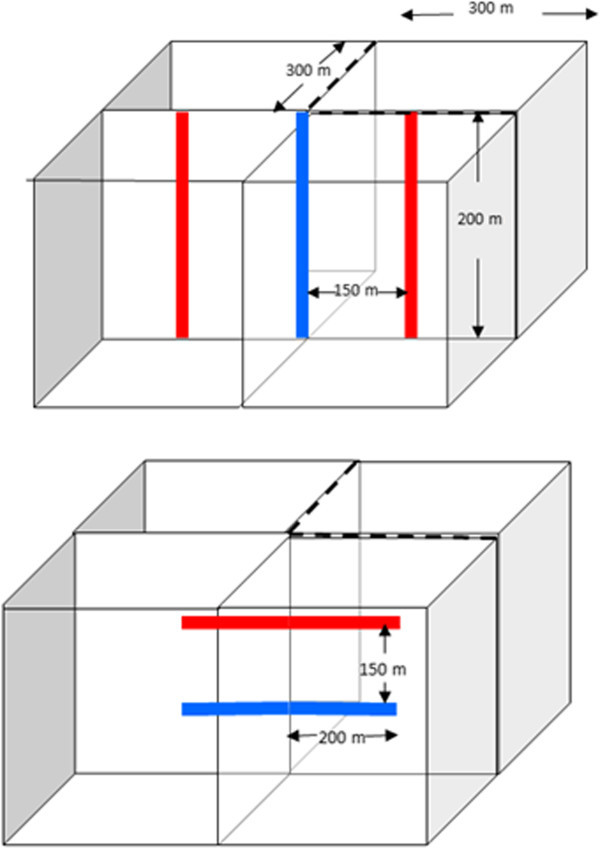
**Alternative Heat extraction schemes. Top: three-point injection scheme with vertical wells.** Bottom: four-point injection scheme with horizontal wells.

The proposed alternative scheme uses horizontal instead of vertical wells. Both schemes are shown in Figure 12. To make these schemes comparable, well length, well separation and total production rate need to be the same in both cases. Two options were considered for the horizontal well scheme: the injection well below the production well, and the injection well above the production well.

The vertical well case uses one injector and 2 producers, which is a 3-point injection scheme. The horizontal well case uses two injectors and two producers, which is a 4-point injection scheme. In both cases the total production is 15 kg/s, the well length is 200 m and the well separation is 150 m. The ¼ modeling domain is used to simulate heat extraction as shown in Figure 12. The only difference between the two cases is in the well orientation. The simulations were done for a homogeneous isotropic reservoir with permeability 1×10^-11^ m^2^ and for a reservoir with one set of vertical fractures similar to one shown in Figure 1, but with the fracture trending along the y axis.

**Figure 13:**
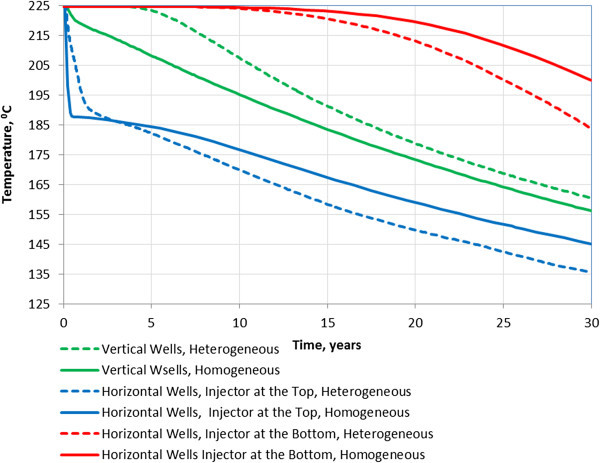
**Average Temperature in the Production Well for Alternative Heat Extraction Schemes.** Heterogeneous case refers to a reservoir with the vertical fractures.

Figure 13 shows the results of the heat simulations for the vertical and horizontal well configurations in terms of average temperature in the production well as a function of time from the beginning of injection.

Figure 13 indicates that the heat extraction for the horizontal wells with the injector at the bottom is significantly better than the heat extraction with the vertical wells. As was expected, placing the injector at the top in the horizontal well scheme negatively impacts the heat extraction as compared to the case with the injector at the bottom. The heat extraction in the case with the injector at the top is worse than in the case with the vertical wells.

The performance of the reservoir with the vertical fractures is slightly better than the performance of a homogeneous reservoir in the case of vertical wells. This is consistent with our previous conclusions (Kalinina et al. 2012a, b).

**Figure 14:**
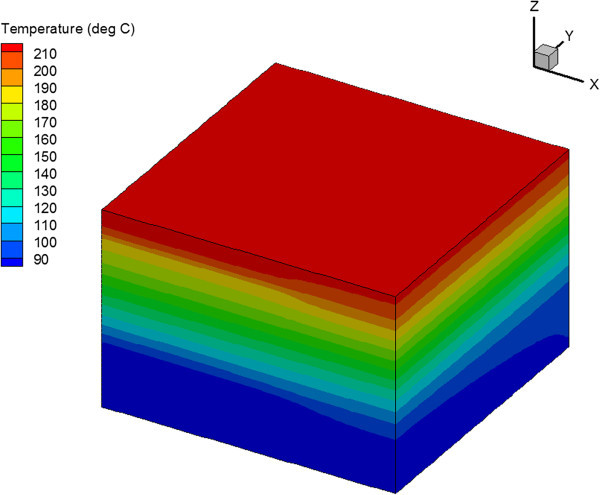
**Temperature Distribution in Homogeneous Reservoir after 30 Years of Injection in the Case of 4-Point Injection Scheme with Horizontal Wells.**

**Figure 15:**
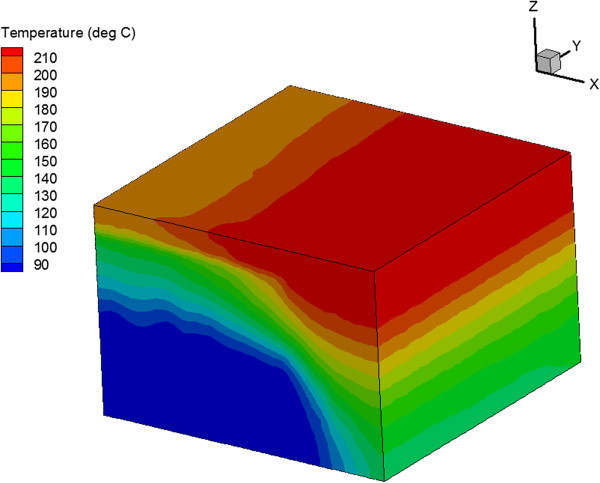
**Temperature distribution in reservoir with vertical fractures after 30 years of injection in the case of 4-point injection scheme with horizontal wells.**

The opposite effect was observed in the case of horizontal wells. The temperature distribution in reservoir is shown in Figure 14 for the homogeneous reservoir and in Figure 15 for the reservoir with one set of vertical fractures. The vertical fractures resulted in percolating temperature front in the vertical direction. This negatively affected the heat extraction in the vertical fracture case compared to the homogeneous reservoir case.

## Conclusions

The fractured continuum model (FCM) provides a tool for mapping the permeability of discrete fractures (either natural or created with hydro-fracturing) onto a regular modeling grid using a continuum approach. The fracture characteristics obtained in the field, such as number of fracture sets, fracture orientation, spacing and aperture, can be used to generate multiple reservoir representations. The FCM can be extended to represent continuous fracture features and fracture intervals with different fracture densities.

The FCM was used to generate permeability fields representing different fracture properties. A 5-point injection scheme was considered for each representation to evaluate the impacts of fracture properties on heat extraction. The following conclusions were made based on the heat transport simulations.

- ▪ The vertical anisotropy is the major factor affecting heat extraction. The heat extraction in the case with fractures dipping 10° (k_xx_/k_zz_ = 33) is only slightly better than in the case with the vertical fractures. The heat extraction in the case of horizontal fractures (k_xx_/k_zz_ = 3,119) is significantly better than in the case of vertical and sub-horizontal (dip 10°) fractures. The improved heat performance in the horizontal fracture case is related to a larger reservoir volume involved in the heat exchange.
- ▪ The average vertical anisotropy is a function of fracture dip. The vertical anisotropy is significant (2 orders of magnitude or greater) only in the case of sub-horizontal fractures with a dip less than 6°. In the case of sub-vertical fractures (dip greater than 60°), the vertical permeability is greater than the horizontal permeability.
- ▪ The heat extraction is further enhanced in the case when there are intervals of high fracture density and low fracture density as compared to the case with constant fracture density with depth. However, the performance is only noticeable better when the vertical permeability in the low density intervals is 2 orders of magnitude lower than in high density intervals. This suggests that creating fracture zones with intact rock between them may be a good way to design an efficient reservoir.
- ▪ The heat extraction with the horizontal wells with the injector at the bottom is significantly better than the heat extraction with the vertical wells. In the case with horizontal wells, the temperature remains constant for the first 15 years of injection and then starts dropping at a rate of 0.7 degree C/year from 15 to 20 years and at the rate of 1.9 degree C/year during the remaining injection time. In the case with vertical wells, the temperature drops nearly linearly from the very beginning of injection at a rate of 2.2 degree C/year.
